# Palmitoylethanolamide: A Natural Body-Own Anti-Inflammatory Agent, Effective and Safe against Influenza and Common Cold

**DOI:** 10.1155/2013/151028

**Published:** 2013-08-27

**Authors:** J. M. Keppel Hesselink, Tineke de Boer, Renger F. Witkamp

**Affiliations:** ^1^Faculty of Medicine, University Witten/Herdecke, Alfred-Herrhausen-Straße 50, 58448 Witten, Germany; ^2^Department of Research and Development, Institute for Neuropathic Pain, Spoorlaan 2a, 3735 MV Bosch en Duin, The Netherlands; ^3^Division of Human Nutrition (Bode 62), Wageningen University, P.O. Box 8129, 6700 EV Wageningen, The Netherlands

## Abstract

Palmitoylethanolamide (PEA) is a food component known since 1957. PEA is synthesized and metabolized in animal cells via a number of enzymes and exerts a multitude of physiological functions related to metabolic homeostasis. Research on PEA has been conducted for more than 50 years, and over 350 papers are referenced in PubMed describing the physiological properties of this endogenous modulator and its pharmacological and therapeutical profile. The major focus of PEA research, since the work of the Nobel laureate Levi-Montalcini in 1993, has been neuropathic pain states and mast cell related disorders. However, it is less known that 6 clinical trials in a total of nearly 4000 people were performed and published last century, specifically studying PEA as a therapy for influenza and the common cold. This was done before Levi-Montalcini's clarification of PEA's mechanism of action, analyzing the role of PEA as an anti-inflammatory agent. We will review in depth these studies, as the results support the effectiveness and safety of PEA in flu and respiratory infections.

## 1. Introduction

Palmitoylethanolamide (PEA) is a food component known for more than 50 years. PEA is synthesized and metabolized by different animal cell types and also present in plants. It exerts a multitude of physiological functions related to metabolic and cellular homeostasis. PEA was already identified in the 50s of the last century as a therapeutic substance with potent anti-inflammatory properties. Since 1970, the anti-inflammatory and other immune-modulating properties of PEA have been shown in a number of placebo-controlled double-blind clinical trials on influenza and common cold. Positive results coincided with the clinical use of PEA in former Czechoslovakia under the brand name Impulsin.

Since 2008, PEA has been marketed as a food for special medical purposes in Italy and Spain, under the brand name Normast (Epitech Srl). Recently, a food-supplement named PeaPure was introduced (JP Russell Science Ltd.). In the USA, PEA is under evaluation as a nutraceutical for inflammatory bowel syndrome (proposed brand name Recoclix, CM&D Pharma Ltd.; Nestlé).

Research on PEA has been conducted since its discovery and over 350 papers are referenced in PubMed describing its physiological properties and role as endogenous modulator as well as its pharmacological and therapeutic effects. PEA is an interesting anti-inflammatory therapeutic substance and might also hold great promise for the treatment of a number of (auto)immune disorders, including inflammatory bowel disease and inflammatory diseases of the CNS. In this paper, we will review the role of PEA as an anti-inflammatory agent and as potential treatment for influenza and the common cold. The main purpose is to highlight and discuss these earliest findings, including the 6 double blind studies in these indications published in the last century using Impulsin. Although nearly forgotten, these findings could offer new insights or perhaps even alternative options in the light of the intense debate around the efficacy and safety of the oseltamivir and zanamivir In the present paper, we will discuss the evolution of knowledge on the anti-inflammatory activity of PEA and its effects in the treatment of respiratory infections.

## 2. The Early Years: Anti-Inflammatory Activity of Egg-Yolk Based on a Lipid-Fraction

The protective and anti-inflammatory effects of PEA can be traced back in the literature to 1939 [1]. The American bacteriologists Coburn and Moore demonstrated in that year that feeding dried egg yolk to underprivileged children living in poor parts of New York City prevented the recurrence of rheumatic fever, in spite of repeated attacks of hemolytic streptococcal infection. 

After 1939, Coburn et al. studied 30 children at a convalescent rheumatic home and prescribed four egg yolks daily. No other change in diet was made and no antibacterial drugs were given. Twenty-two of these children contracted 24 serologically positive group-A streptococcal infections, but none showed clinical evidence of rheumatic recurrences. This was in sharp contrast to previous experience in the convalescent home where rheumatic recurrences had been frequently seen each year [2].

Subsequently in 1954, Coburn and colleagues were also the first to report a phospholipid fraction prepared from egg yolk that showed antiallergic activity in an assay in the guinea pig [3]. 

The antiallergic factor of egg yolk was then purified by Long and Martin in 1956 in such a way that it was clear that this factor showed a biological and chemical similarity to a preparation obtained earlier in 1950 from peanut and what appeared to be a closely related substance described as “vegetable lecithin” [4, 5]. 

The birth year of PEA was 1957. Kuehl Jr. and coworkers reported to have succeeded in isolating a crystalline anti-inflammatory factor from soybean lecithin and they identified it as *N*-(2-hydroxyethyl)-palmitamide [6]. They isolated the compound also from a phospholipid fraction of egg yolk and from hexane-extracted peanut meal. The product obtained was tested positively in a local passive joint anaphylaxis assay in the guinea pig. When applying their isolation procedure to soybean lecithin, they obtained a partially purified fraction from which the homogeneous factor was obtained by crystallization from cyclohexane. The crystalline material had a melting point of 98-99°C and was described as neutral, optically inactive, and possessing the chemical formula C18H37O2N. 

Hydrolysis of the factor resulted in palmitic acid and ethanolamine and thus the compound was identified as *N*-(2-hydroxyethyl)-palmitamide. In order to close the circle of isolation and identification, Kuehl et al. were able to synthesize the compound by reflux in ethanolamine with palmitic acid according to a well-known procedure described in the chemical literature of that time. Kuehl et al. further analyzed the anti-inflammatory activity of a series of derivatives of PEA and could prove that the basic moiety of the molecule was responsible for its anti-inflammatory activity. The nature of the acid group appeared to them to be of no importance because in addition to ethanolamine itself, *N*-(2-hydroxyethyl)-lauramide, S-(2-hydroxyethyl)-salicylamide, and *N*-(2-hydroxyethyl)-acetamide all had potent anti-inflammatory properties. These pharmacological properties of the ethanolamine-derivatives appeared to be quite specific since other homologs did not show a biological response in the assay. 

## 3. The Protective Effects of “Proto-PEA” in Streptococcal Infections

 Coburn was dedicated to find the cause and prevention of rheumatic fever [7]. He presented his hypothesis that eggs contained an important protective factor against infection, especially in rheumatic fever, in 1960 in the Lancet [2]. He argued that

- inadequate nutrition is part of a poor environment;
- rheumatic-fever children usually lack sufficient eggs in their diets;
- the escape from poverty is followed by an increase in the consumption of eggs and a decrease in the incidence of rheumatic fever;
- supplementation of children's diets with egg yolk or certain fractions thereof is followed by decreased rheumatic susceptibility; and
- there is a fraction of egg yolk, which in extremely small amounts has been found to have high antiallergic activity in laboratory animals.

Coburn described his field studies in great detail [2]. Some of these findings are summarized below. 

In Field study number 1, *n* = 89, rheumatic boys and girls living at home in New York City all received egg-enriched food; no prophylactic drugs were given. Sixty children had extra eggs during winter and spring months, and 29 served as “controls.” Results were as follows: of the 29 children on their normal diet (with many nutritional deficiencies) 11 had a recurrence. Of the 35 children whose normal diet was enriched with two eggs daily, a quart of milk, meat, butter, and halibut-liver oil, 3 had a recurrence. Of the 25 children whose normal diet was reinforced only with powdered egg yolk (equivalent to six eggs daily), only 1 had a recurrence.

Field study number 2, *n* = 56, was a two-year study into the effect of giving egg-yolk powder (equivalent of four egg yolks daily) to rheumatic children for three to four weeks after they developed hemolytic (group A) streptococcal pharyngitis. No other treatment was given during this period. Results were as follows: of 28 receiving the supplement, only 1 showed fresh rheumatic activity, whereas among 28 “controls,” receiving no supplement, 10 children had fresh rheumatic activity.

Field study number 3, *n* = 40, was a one-year study in which about 40 rheumatic children (with many dietary deficiencies) received a daily supplement of only the protein fraction from four egg yolks. Results were as follows: study was discontinued because of too many rheumatic recurrences.

Field study number 4, *n* = 45, was a four-year study (Chicago, period 1952–1956) in which a normal (nutritionally deficient) diet of rheumatic children was reinforced with egg-yolk alcohol-soluble material (A.S.M. from Wilson Laboratories). No other changes in their inadequate diets were made; no sulphonamides, antibiotics, or other significant drugs were administered. Forty-five highly susceptible rheumatic children received this supplement throughout the school year from September to July. The equivalent of 3 egg yolks was consumed, in the form of an elixir taken twice daily. All but one of these rheumatic children was under fifteen years of age. Results were as follows: a minimum of 17 attacks was expected among them after streptococcal infections, but only 5 occurred. 

Coburn concluded that “The data obtained under these various conditions, both in New York and a decade later in Chicago, were found to be statistically significant.” However, he himself acknowledged that all studies had methodological weaknesses [2].

Coburn discussed various experimental findings around that time supporting the idea that there is at least one anti-inflammatory substance present in egg-yolk alcohol-soluble material, which was not present in the protein or acetone-soluble material [5, 8]. The anti-inflammatory activity was confirmed by different groups, for instance, by measuring joint and skin lesions in either the Arthus or tuberculin reaction. Various models were used and all results were supportive of Coburn's observations. The anti-inflammatory compound clearly was part of the lipid fraction of the egg and not the protein-water fraction.

## 4. Acceptance of PEA's Anti-Inflammatory Effects

Already in 1965 the anti-inflammatory activity of PEA seemed to be quite well known in the scientific community. Amongst others, Bachur, from the Laboratory of Clinical Biochemistry and Experimental Therapeutics Branch, National Heart Institute, National Institutes of Health, Bethesda, MD, USA, and colleagues referred extensively to the findings of Kuehl et al. (1957): “Kuehl et al. have previously reported the isolation of PEA, as a naturally occurring anti-inflammatory agent, from egg yolks. PEA was known to occur in nature and to have pharmacological activity” [9].

The group of Bachur analyzed the content of PEA and found it to be present in several tissues of the rat and guinea pig. The amounts found in liver were quite variable, but PEA was consistently found in brain, liver, and muscle tissue and was not detected in other tissues examined. Around that time, the anti-inflammatory action of PEA could also be demonstrated in a classical anti-inflammatory model, the carrageen-induced edema model [10].

In the beginning of the 70s, the modifying effects of PEA on immunological reactions were well established [11]. Perlik et al. summarized [12] that “It has been shown that *N*-(2-hydroxyethyl)-palmitamide (PEA) can decrease the intensity of several inflammatory and immunological processes.” 

However, between 1958 and 1969 the interest in this compound had apparently decreased, as the same authors stated that “Recently the interest on biological properties of PEA has been revived because of its capacity to increase nonspecific tolerance to several bacterial toxins.”

## 5. PEA: Anti-Influenza and Anticommon Cold

New interest arose by the end of the 60s, due to the fact that SPOFA United Pharmaceutical Works brought PEA on the market in 300 mg tablets under the brand name Impulsin to treat influenza and common cold. Different clinical trials supported the effectiveness and safety of PEA for this indication. Most probably the PEA in Impulsin was not specifically formulated but details are not available.

In the period of 1969–1979, the results of a total of 5 trials in adults and one trial in children were published. All of these were double-blinded and placebo-controlled.

In the 1974 paper by Masek et al., the first two double-blind controlled trials were described with a total of 1344 healthy subjects randomized (see Table 1: Masek 1972a and Masek 1972b). There were in total 40 dropouts during the studies, meaning that 1304 subjects completed the trials. The goal of these two trials was to evaluate the prophylactic and treatment efficacy of Impulsin in upper respiratory tract infections. Both trials ended in February 1973 [13].

The first trial (Masek 1972a) was a treatment trial; 468 employees of the Skoda car factory were randomized in this trial; of these 444 were completers, available for analysis. The employees had to register the following symptoms: temperature of 37.5°C or higher, headache, sore throat, myalgia, nasal stuffiness or discharge, productive or dry cough, malaise, and fatigue and had to make a clear impression of being sick. Dosing was 600 mg Impulsin three times daily during 12 days (total daily dose 1800 mg PEA).

The second trial was a prophylactic trial; 918 volunteers between 18 and 20 years of age from an army unit were included, and 899 completed the trial period. In this trial, medical personnel registered the complaints during a period of 8 weeks. The treatment schedule was 600 mg PEA three times daily for the first 3 weeks, after which a continuation phase started based on a single dose of 600 mg once daily for 6 weeks. 

The results from the first trial showed that patients receiving PEA had a lower number of episodes of fever, headache, and sore throat, compared with placebo patients (18 versus 33). PEA had less effect on symptoms such as nasal stuffiness, discharge, and cough. The episodes of fever and pain were significantly reduced by 45.5% in the PEA group compared with the placebo group (*P* < 0.05). The beneficial effect of PEA was apparent from the second week of the trial. The total number of sickness days was also significantly reduced in the PEA group. In the prophylactic trial, Masek 1972b, the incidence of disease in the PEA group was 40% lower at week 6, and 32% lower at week 8 compared to placebo (*P* < 0.0005).

In order to verify the conclusions, 3 more trials in soldiers were conducted. Soldiers were selected as they were housed close together. In the period of 1973–1975, these new trials were initiated (Kahlich 1973, 1974, and 1975 in Table 1) and the results were reported in 1979 by Kahlich et al. [14]. Due to the positive effects, it was felt to be unethical to randomize 1 : 1 and in the last two trials a different randomization schedule was selected, in order to dose the majority of volunteers with Impulsin (2 : 1). The authors compared the incidence of clinical endpoints and the titers of influenza viruses between both the PEA and placebo groups. In all three trials, the soldiers in the PEA group had significantly less symptoms and were less often diagnosed as flu patients (see Table 1). 

The evaluation of results according to morbidity, regardless of etiology, showed a significant reduction in acute respiratory diseases (ARD) after administration of PEA. In the 1973 trial (901 volunteers), 22.7% of ARD cases were found in the PEA group compared to 34.4% in the placebo group (*P* < 0.0002). The relevant values in the 1974 trial (610 volunteers) were 19.7% and 40.7% (*P* < 0.002) and in the 1975 trial (353 volunteers) 10.6% and 28.8% (*P* < 0.004) [14]. 

In all studies, Kahlich et al. studied serology in order to document the influenza strains. The codes of these strains are described below; however, the nomenclature is outdated. A fourfold increase in the antibody titer was taken as evidence for infection. 

In the 1973 study, serum was obtained from 358 persons. 6.9% of the subjects experienced influenza A 2 E in the PEA group and 18.7% of the subjects in the placebo group (*P* < 0.005). The manifestation rate (MR), expressing the proportion of sick persons out of all sensitive subjects with serologically proved infection, was 15.4% in the PEA group and 44.9% in the placebo group (*P* < 0.0002).

In the study of 1974, sera of 108 subjects were analyzed. In the PEA group 3.8% of the subjects suffered from the influenza B Hong-Kong and 21.4% of the subjects in the placebo group (*P* < 0.01). The MR was 14.3% in the PEA group and 57.1% in the placebo group (*P* < 0.001).

In the study of 1975, with serum gathered from 212 subjects, only 4.3% of the subjects in the PEA group had influenza A Port Chalmers and 7% of the subjects in the placebo group (nonsignificant difference). The incidence rate of influenza A 2 England was 15.4% in the PEA group and 44.9% in the placebo group (*P* < 0.0002). 

All these clinical trials pointed in the same direction that PEA has clear treatment effects in respiratory infections, can be used as influenza-prophylaxis, and is safe in its use. Side effects were not reported, and Kahlich et al. explicitly stated that “No side effects were registered after several years of clinical testing of Impulsin in military and civilian communities.” Kahlich et al. also pointed out that the effects of PEA had a clear advantage over vaccines and antiviral compounds such as amantadine, due to the optimal balance of efficacy and side effects of PEA. They also stated that the ease of application of PEA offers the possibility to have a quick therapeutic answer ready in case of a flu epidemic, especially in cases of a mismatch between circulating strains and the recommendations from WHO. 

A last placebo-controlled study with PEA in children aged 11 to 15, examining the incidence of acute respiratory tract infections, was performed in January 1976 [15]. 457 children were included and divided into 2 groups; 64 children dropped out. In the PEA group, 169 children completed the study who received 300 mg PEA 2 times daily with an interval of 6 hours. The placebo group included 224 children receiving 2 placebo tablets following the same regime as the PEA group.

Blood samples were taken before the study and 8 weeks later in 65% of all children. After 8 weeks, children treated with PEA had 15.7% fewer acute respiratory tract infections than the control group. In children from 11 to 13 years of age, the difference was even more pronounced: 25.5%. Due to the short duration of the intake of PEA and the absence of epidemic influenza during the trial period, the differences between both groups were not very large, and thus no significance was reached.

Taken together, in the period between 1972 and 1977 in total 3627 patients and volunteers completed 6 different placebo-controlled double-blind trials of which 1937 received PEA up to 1800 mg/day. Relevant side effects were not reported and especially the trials conducted during the flu season demonstrated a treatment, as well as a prophylactic effect. The last study in children was not significant due to the fact that during the study period no influenza epidemic occurred.

## 6. PEA: Anti-Inflammatory Actions and Its Mechanism of Action via PPAR-Alpha Agonism and Other Targets

Since a decade, NAEs, both as saturated fatty amides (such as PEA) and as poly-unsaturated forms, are found to play an important physiological role in the modulation of immune reactions in a number of autoimmune disorders via a number of different receptors. For instance, Celiac disease is an autoimmune disorder of the small intestine caused by a reaction to gliadin, a gluten protein found in wheat. Most likely endocannabinoids play here an important modulating role [16]. Anandamide and PEA concentrations in Celiac disease were significantly elevated (100% and 90%, resp.) during the active phase, as was the number of CB1 receptors. The levels returned to normal after remission with a gluten-free diet [17]. This clearly can be interpreted as the activation of a self-repairing mechanism.

In an elegant study on the anti-inflammatory and pro-apoptotic activities of anandamide, it was shown that it can inhibit tumor necrosis factor-*α*-induced NF-*κ*B activation [18]. The NF-*κ*B inhibitory activity of anandamide was independent of CB1 and CB2. Structure-activity relationships demonstrated that analogs with saturated fatty acyl groups were more active than unsaturated analogs. Saturated acylethanolamides such as PEA therefore offer a new opportunity to modify chronic inflammation in autoimmune disorders.

For a long period of time after the first description of PEA, its mechanism of action remained unsolved, and this led to a weaning interest in the compound after the set of publications on the efficacy and safety of PEA in respiratory infections and flu (in the period of 1970–1980). New interest in the mechanism of action of PEA emerged only after the work of the Nobel laureate Professor Rita Levi-Montalcini, who published a seminal paper in 1993, opening the door to a new understanding of PEA's anti-inflammatory and analgesic actions [19, 20]. Starting with her work, it became clear that PEA regulates many pathophysiological processes, and PEA has since been found to be effective in a number of animal models for inflammation, neuroinflammation, neurotoxicity, and chronic pain. Levi-Montalcini highlighted the importance of activation of inflammatory cascades via the activation of nonneuronal cells, such as the mast cells [21]. PEA reduces mast cell migration and degranulation and reduces the pathological overactivation of these cells [21]. Mast cells shift from activated immune- to resting phenotypes under influence of PEA. PEA further reduces the activity of the proinflammatory enzymes, cyclooxygenase, and endothelial, and inducible nitric oxide synthases. PEA has an additional number of other pharmacological and physiological properties, such as its affinity for the novel orphan cannabinoid receptors GPR55 and GPR119 and for the vanilloid receptor TRPV1, as well as for the nuclear peroxisome proliferator-activated receptor-*α* (PPAR-*α*) [22–25]. These are probably the most relevant mechanisms of action of PEA related to immunopathology. 

## 7. Metabolism of PEA

### 7.1. Synthesis

In the body, PEA is synthesized from palmitic acid (C16:0), which is the most common fatty acid in animals and a product of normal fatty acid synthesis. Palmitic acid is also present in many foodstuffs including palm tree oil, meats, cheeses, butter, and dairy products. Synthesis of PEA takes place in membranes of various cell types and involves different steps and partly parallel pathways. The most studied pathway goes via N-palmitoyl-phosphatidyl-ethanolamine, which belongs to the class of N-acylphosphatidylethanolamines (NAPEs). NAPEs in general are present in phospholipid membranes and function as stable precursors and source of their respective NAEs. Palmitic acid is incorporated from the sn-1 position of a donor phospholipid like phosphatidylcholine and transferred to an ethanolamine phospholipid, for example, phosphatidylethanolamine, which is catalysed by a Ca^2+^-dependent N-acyltransferase [26–28]. Next, free PEA can be generated by a NAPE-hydrolyzing phospholipase D (NAPE-PLD). However, recent studies also demonstrate the presence of NAPE-PLD-independent multistep pathways to form NAEs from NAPE [28]. 

An alternative pathway involves formation of NAEs from N-acylated plasmalogen-type ethanolamine phospholipid (N-acyl-plasmenylethanolamine) through both NAPE-PLD-dependent and independent pathways [28]. In general, tissue patterns of NAEs are considered to reflect the local availability of their precursor fatty acids in the phospholipid membranes, which are amongst others diet-related [29]. However, in case of PEA, tissue levels hardly seem to be influenced by variation in intake of dietary fatty acids, except in the small intestine where dietary fat results in decreased levels of PEA and other NAEs [27, 30]. Several studies indicate that free PEA levels increase during inflammation [27, 30, 31]. Concentrations of PEA in tissues and plasma have been published in various papers, as recently reviewed by [27, 30, 31]. In humans, PEA plasma concentrations are subject to considerable variation during the day [32].

### 7.2. Breakdown

Like with other NEAs, endogenous PEA is produced on demand and acts locally. Tissue levels are tightly regulated through a balance between synthesis and breakdown. The primary degrading enzyme is fatty acid amide hydrolase (FAAH, now also known as FAAH-1), localised on the endoplasmatic reticulum [33]. A second FAAH enzyme, now called FAAH-2, was found in humans, located on cytoplasmic lipid droplets [33, 34]. Recently, a third NAE hydrolysing enzyme, N-acyl ethanolamine-hydrolyzing acid amidase (NAAA) has been identified [28]. In the cytosol, fatty-acid binding proteins and heat-shock proteins may serve as carriers for PEA to their degrading enzymes [27].

## 8. PEA Anti-Influenza Activity: Decrease of Proinflammatory Cytokines

After an infection with the influenza virus, the immune system reacts by an increased production of many cytokine-patterns. One pattern is related to a proinflammatory response and a second one to an antiviral response. Infections with virulent influenza viruses together with an aberrant and excessive cytokine production are linked to increased morbidity and mortality [35]. Increased production of specific inflammatory cytokines, such as the tumor necrosis factor (TNF)-*α*, interleukin- (IL-) 1, IL-6, and IL-10, is characteristic during an influenza infection [36]. More virulent viruses are also associated with rapid and sustained induction of inflammatory cytokines and such an early dysregulation of the host response is seen as contributing to the severity and outcome of the infection [37, 38]. The increased production of proinflammatory cytokines, hypercytokinemia, is thus a clear player in the disease progression and death of patients infected by influenza viruses [39, 40]. Recently, it was demonstrated that highly elevated levels of serum IL-6 and IL-10 in A (H1N1) patients may also lead to disease progression [41].

Overactive and nonfunctional hyper induction of proinflammatory cytokines might therefore play a key role in the symptomatology and may lead to increased morbidity and mortality. PEA is widely known for its anti-inflammatory activity and to date more than 60 PubMed indexed papers discuss this property of PEA. Its inhibitory action on TNF-alpha secretion is sufficiently documented [42]. But PEA has a much wider modulating effect on interleukins. For instance, recently PEA was shown to significantly attenuate the degree of intestinal injury and inflammation and to inhibit proinflammatory cytokine production (TNF-*α*, IL-1*β*), adhesion molecules (ICAM-1, P-selectin) expression, and NF-*κ*B expression [43]. PEA also significantly decreases inflammation caused by ischemia-reperfusion injury, a pathological state characterized by a strong enhanced interleukin-cascade [44]. As PEA downmodulates a number of proinflammatory cytokines, this could very well be the reason for the decreased influenza and common cold symptomatology in individuals treated with PEA.

## 9. Conclusions and Therapeutic Perspective

 Over 350 papers have been referenced in PubMed in the last 50 years describing the physiological properties of PEA and its pharmacological and therapeutical profile. PEA has a broad spectrum of biological targets and target molecules, among which are PPAR-alpha, TRPV1, and orphan receptors such as GPR-55.

This review on the role of PEA as an anti-inflammatory agent and as a therapeutic agent for influenza and the common cold discusses 6 clinical trials in a total of nearly 4000 patients and volunteers where PEA's effectiveness and safety for the treatment in these indications was shown. Furthermore, since the focus on respiratory inflammation and flu between 1971 and 1980, PEA has also been extensively tested in a great variety of animal models for a number of other indications, such as central and peripheral neuropathic pain, pain in osteoarthritis, traumatic brain injury, multiple sclerosis, amyotrophic lateral sclerosis, Alzheimer's disease, irritable bowel disease, interstitial cystitis, and other visceral pain states. Consistently, the effective dose range has been between 10 and 30 mg PEA/kg bodyweight [45, 46]. Since the work of Levi-Montalcini in the 90s of the twentieth century, results of around 40 clinical trials in chronic pain have been reported. However, the majority of these results were reported in Italian and Spanish medical journals [37]. Since 2008, an increasing number of clinical data have been reported in English literature and results support its use in indications such as sciatic pain and related neuropathic pain disorders. As PEA clearly plays a fundamental role as a protective and restorative modulating lipid precursor, its clinical role is currently further evaluated in a variety of disorders such as inflammatory bowel disorder, central neuropathic pain in spinal cord disorders, various eye disorders such as glaucoma and degenerative retina disorders, multiple sclerosis, amyotrophic lateral sclerosis, and Alzheimer's disease. 

Given the results of 6 clinical trials in flu and the common cold, seen in the context of the serious criticism on the efficacy and safety of oseltamivir and zanamivir, PEA should be reconsidered by clinicians as a new treatment modality for the flu and respiratory infections due to its documented efficacy and more importantly its very benign side effect profile. Furthermore, oseltamivir and zanamivir are known to induce resistance; PEA has a very low likelihood of inducing resistance due to its mechanism of action. Finally, the ease of application of PEA offers the possibility to have a quick therapeutic answer ready in case of a flu epidemic, especially in cases of a mismatch between circulating strains and the recommendations from WHO.

## Figures and Tables

**Table 1:** Incidence of endpoints between both the PEA and the placebo groups.

Study (year)	PEA (*n*)	Placebo (*n*)	% Protection	Significance (*P*)
Masek (1972a) [14]	223	221	45%	<0.05
Masek (1972b) [14]	436	463	32%	<0.0005
Kahlich (1973) [14]	436	465	34%	<0.0002
Kahlich (1974) [14]	411	199	52%	<0.002
Kahlich (1975) [14]	235	118	59%	<0.004
Plesnik (1977) [15]	196	224	16%	NS
